# Exploration of children’s value patterns in relation to environmental education programmes

**DOI:** 10.3389/fpsyg.2023.1264487

**Published:** 2023-11-13

**Authors:** Tim Kelly, Thijs Bouman, Simon Kemp, Franka Wijngaarden, Randolph C. Grace

**Affiliations:** ^1^School of Psychology, Speech and Hearing, University of Canterbury, Christchurch, New Zealand; ^2^Department of Psychology, Faculty of Behavioural and Social Sciences, University of Groningen, Groningen, Netherlands

**Keywords:** biospheric values, pro-environmental behavior, children, peers, environmental education

## Abstract

During childhood we begin to develop values, including valuing the natural environment (biospheric values). Although biospheric values are believed to provide the foundation for pro-environmental behavior throughout the course of one’s life, little research has investigated these values in children. The present study aimed to investigate the relationships between children’s endorsement of biospheric values, their pro-environmental behaviors, and their perception of their friends’ and peers’ endorsement of biospheric values. Moreover, we investigated whether these values and behaviors, as well as the hypothesized relationships, were affected by educational programmes that were already implemented at schools. The results showed that children generally strongly endorse biospheric values, and that biospheric values were positively related to some personal and group pro-environmental behaviors. The study also found that, as in previous research with adults, the participants believed that their friends and peers endorsed biospheric values significantly less than they themselves did. Environmental educational programs were partially effective in reducing the participants’ underestimation of their friends’ biospheric values and increased the likelihood of some group pro-environmental behaviors. Our findings highlight the need for further research to investigate the effects of group pro-environmental behaviors and the perception of group values.

## Introduction

The pro-environmental behavior of individuals makes an important contribution to global environmental sustainability (Vlek and Steg, 2007; Schill and Godefroit-Winkel, 2019; Intergovernmental Panel on Climate Change, 2022). As a result, considerable research has been dedicated to understanding the basis for everyday environmental behaviors. One such field of research is the study of basic human values and their relationship to environmental behaviors (Stern and Dietz, 1994; Dietz et al., 2005; Steg and de Groot, 2012; Gatersleben et al., 2014; Bouman et al., 2021; Steg, 2022). Specifically, research has indicated that individuals’ pro-environmental behaviors are typically grounded in their personal, as well as their groups’, biospheric values, which reflect the degree to which the individual or group cares about nature and the environment. In this article, we examine whether children’s values and pro-environmental behaviors are affected by their participation in existing group environmental education programmes and investigate whether these programmes influence children’s perception of their friends’ and peers’ values.

Basic human values, including biospheric values, are broad, stable, and desirable goals that function as guiding principles on which people evaluate their actions and decisions (Rokeach, 1973; Schwartz, 1994; Maio and Olson, 1998; Brosch and Sander, 2015; Dietz, 2015). The more a person endorses a value, the more influence this value has on the person’s decision making and actions. In particular, individuals are more likely to take actions that have relatively many benefits, and little costs, for more strongly endorsed values. Understanding biospheric values in childhood is critical because values are generally considered to be less fixed at this age but then stabilize over time (Stern et al., 1995; Schwartz et al., 2001; Thøgersen and Ölander, 2002; Bilsky et al., 2011; Manfredo et al., 2017). Even though people begin to start valuing the natural environment from an early age (Thøgersen and Ölander, 2002; Zeiske et al., 2021), surprisingly few studies have investigated these childhood biospheric values in any depth.

Valuing the natural environment (biospheric values) is a significant predictor of environmental behaviors (Stern and Dietz, 1994; Steg et al., 2014; Bouman et al., 2018, 2021; Lee et al., 2022; Steg, 2022). The more an individual endorses biospheric values, the more likely this person generally is to engage in pro-environmental behavior (Stern et al., 1995; Thøgersen and Ölander, 2002; Bouman et al., 2021; Steg, 2022). Indeed, biospheric values have been shown to be positively related to a wide range of pro-environmental behaviors, including recycling (Balunde et al., 2019), turning off the lights when leaving the room (Zeiske et al., 2021), choosing not to eat meat (van der Werff et al., 2013), spending less time in the shower (van der Werff et al., 2013; Zeiske et al., 2021), and environmental activism (Balunde et al., 2019). With this in consideration, our first hypothesis (H1) is that children’s biospheric values will be positively related to their pro-environmental behaviors.

Although a small number of studies have examined the influence of perceived peer values on adult’s biospheric values (Hanel et al., 2018; Bouman et al., 2020; Wang et al., 2021), we are unaware of any research that has examined this influence in children, despite childhood being a time when peers are highly influential (Harris, 1995; Benish-Weisman et al., 2022). It is likely that a person’s biospheric values are influenced by their understanding of what their friends and peers value. Past research has shown that adults’ perception of what their peers value is predictive of their own individual values, including their biospheric values (Bouman et al., 2020; Wang et al., 2021). Although this has not been investigated in children, recent empirical research has shown that children influence each other’s self-transcendence values (caring about other people and nature), and that this effect is most prominent in early adolescence (Benish-Weisman et al., 2022). Friends and peers are likely to be most influential during childhood because children increasingly seek to define their identity during their pre-teen and teenage years (Harter et al., 1996). Social comparison and imitation may cause a homophily effect, whereby the perceived values of the social group become more strongly adopted by the individual group members as a way of increasing their sense of belonging within the group (Brechwald and Prinstein, 2011). Peer feedback can influence this identity development (Hergovich et al., 2002) and children may seek to emulate the behaviors and values of the peers within their social group, if doing so enables them to develop an intrinsically rewarding self-identity (Abrams and Hogg, 1990). Given that previous research has found that children strongly endorse biospheric values (Thøgersen and Ölander, 2002; Zeiske et al., 2021) and that children are likely to be influenced by what they perceive other children to value (Benish-Weisman et al., 2022), we hypothesize (H2) that children’s personal biospheric values will be positively related to their perception of their friends’ and peers’ biospheric values.

Although children are likely to be influenced by their friends’ and peers’ values, their perception of what their friends and peers value may not be accurate. This has already been shown to be the case for childhood environmental behavior: people tend to underestimate or overestimate their peers’ behavior (Brechwald and Prinstein, 2011) and a study by Long et al. (2014) found that New Zealand adolescents underestimated their friends’ pro-environmental behavior (recycling and not littering) and to a greater extent their wider peers’ pro-environmental behavior. Bouman et al. (2020) found that adults underestimate their peers’ environmental values, but we are unaware of any research that has examined this possibility in school-age children. A problem is that children may not have many opportunities in everyday life to display their biospheric values to others. In this regard, misperception, particularly underestimation, of other people’s values may be expected. Therefore, if children underestimate their friends’ and peers’ biospheric values as hypothesized (H3), this may prevent them from strongly endorsing biospheric values themselves.

Our fourth hypothesis (H4) is that participation in group environmental educational programmes will increase the individual’s perception of their peers’ biospheric values. Interventions that correct the potential underestimation of friends’ and peers’ biospheric values may be a useful way to change environmental behavior. In this regard, if school children are placed in contexts that cause them to engage in environmental dialog and behaviors with each other, or to observe each other’s biospheric values in action, this may enable them to develop a clearer understanding of their peers’ biospheric values, which may cause them to endorse biospheric values more strongly themselves, since it is widely accepted by their peer group (Brechwald and Prinstein, 2011). Contexts that may induce this effect include, for instance, group education programmes that explore the impact of humans on the environment, working with peers on community environmental projects, participation in pro-environmental fora, and group nature-based activities.

## Method and results

### Study 1 (New Zealand)

#### Study 1 method

##### Participants and design

In New Zealand we collected data from children who were participating in a group environmental education intervention based on the New Zealand Qualifications Authority (NZQA) learning standard titled “*AS90813: Demonstrate understanding of how different personal values have implications for a sustainable future*.” The learning content for this intervention was developed by the researchers as part of a separate study and used by all participating schools. The intervention was preceded by a survey and followed by surveys immediately after the intervention and 3 months after the intervention. The intervention was delivered by teachers in five different schools of varying socio-economic status across New Zealand, and typically took between 16 to 20 h to deliver. Four of the schools were rural, and one was urban.

The learning materials for this education course required participants to explore Schwartz’s value system (Schwartz, 1992, 2017) and to identify their own value priorities. The impact of different personal values on the environment was explored, taking various personal and cultural perspectives. The intervention was based in part on Maio and Olson’s (1998) finding that values can change when individuals are required to explicitly consider why they hold such values. The learning intervention was mainly delivered in a discursive format, during which the participants would become more aware of each other’s personal values.

The surveys were administered online via the Qualtrics platform. After completing each survey, the respondents were immediately returned a summary of their key survey findings (mean basic human values and environmental behavior scores) so that they could better understand their own values and behaviors and refer to these in their subsequent learning.

Ethics approval for the New Zealand investigation was obtained from (the University of Canterbury’s Educational Research Human Ethics Committee). Consent to be surveyed was required from the participants, their parents/guardians, their teacher, and their school. Students were required to provide their name and email address on the survey so that (a) we could return to them a summary of their individual results after each survey, and (b) so that individuals’ data from surveys could be paired to examine the intervention effect. By analyzing standard deviations across individual constructs, and by examining responses to reverse coded variables, twelve disengaged cases were identified and removed from the study. As a result of this process and the loss of some participants over the course of the investigation, different numbers of responses were collected at each survey point. A criterium for inclusion in the investigation was that the first survey be completed, along with either the second or third survey. The final number of cases analyzed were 77 (T1: pre-intervention), 75 (T2: post-intervention), and 63 (T3: 3 months post-intervention). We also attempted to collect data from control schools/classes, but the completion rates at each timepoint were insufficient for analysis. The average age of participants was 14.7 years, with an age range of 14 to 16 years. In the first sample, thirty-four of these participants were female and forty-four were male.

##### Measures

The measures were presented in the survey in the following order:

###### Personal biospheric values

Bouman et al. (2018) Environmental Portrait Values Questionnaire (E-PVQ) was employed to measure participants’ self-transcendent (biospheric and altruistic) and self-enhancing (egoistic and hedonic) values. A seven-point scale from “strongly disagree” to “strongly agree” was employed. For the four biospheric values indicators, the internal reliability (alpha) of the construct was larger than 0.86 at each survey point.

The full basic human values scale was followed by a short personal values scale. Further in the survey, the participants were asked to also rate their peers’ and parents’ values’, so it was necessary to incorporate a short scale for this, to avoid making the survey too arduous for school children. The short values scale was titled “How important are these things to you?” and consisted of a single indicator representing each of biospheric (“It is important to me to take care of, and live in harmony with, nature”), altruistic (“It is important to me to help others and that others are treated fairly”), egoistic (“It is important to me to have wealth, possessions, influence, and status”), and hedonic (“It is important to me to have fun and have a good time”) values, which we deemed to encompass the conceptual breadth of each value. Scoring was on a similar seven-point scale to the full scale and, as for the full scale, the participants were instructed to vary their scoring for each indicator as much as possible. At each survey point we found that the short scales measuring the participants’ own values were highly correlated with the corresponding long scales (*r*s > 0.76) and can thus be considered valid indicators of basic human values for this purpose. For the sake of consistency, and to be able to compare personal values with perceived values of peers and parents, only the findings from the short value scales are presented in this article.

###### Personal pro-environmental behavior

The self-reported frequency of five environmental behaviors was measured: “Drop your litter on the ground instead of disposing of it properly,” “Switch off electrical appliances instead of leaving them on stand-by,” “Leave the tap running when brushing your teeth,” “Recycle your waste that can be recycled,” and “Spend less time than you’d like in the shower, to save electricity or water.” These behaviors were specifically chosen because they are relevant to children. The first and third of these were reverse coded, and an index of the five behaviors was created (see Table 1 for M and SD at each survey point). These self-reported pro-environmental behaviors were measured on a five-point Likert scale from “Never” to “Always.”

**TABLE 1:** Values and behaviors at each survey point (NZ).

	Pre-intervention		Post-intervention		3 months post-intervention	
	**Mean**	**SD**	**Mean**	**SD**	**Mean**	**SD**
Personal biospheric values^1^	5.43	1.12	5.43	1.30	5.42	1.16
Perceived peers’ biospheric values^1^	4.67	1.32	4.76	1.08	4.79	1.07
Personal PEB frequency^2^	3.59	0.60	3.64	0.67	3.52	0.69
Environmental dialog with peers^1^	3.40	1.44	3.72	1.69	3.98	1.63
Environmental dialog with parents^1^	4.09	1.71	4.24	1.65	4.12	1.77
Challenge peers harming environment^1^	5.06	1.47	4.81	1.71	4.77	1.63
Help environment when with peers^1^	4.42	1.63	4.51	1.65	4.57	1.57

^1^Out of seven. ^2^Out of five. PEB, pro-environmental behavior.

The next section of the survey concerned measurement of peer-related factors. Participants were given the following definition of peers: “Your ‘peers’ are the people at school who are around the same age as you. When answering the questions, just think about people your age in general.” All of the following responses were measured on a seven-point scale from 1 “extremely unlikely” to 7 “extremely likely.”

###### Peers’ biospheric values

The short values scale described above was presented again, but with peers as the subject, e.g., “It is important to your peers to take care of, and live in harmony with, nature” in response to the question “What is important to your peers?” (see Table 1 for M and SD at each survey point). The differences between the perceived group values at each survey point, and the differences between the participants’ personal biospheric values and their perception of their peers’ biospheric values, were determined.

###### Peers’ pro-environmental behavior

The frequency of environmental dialog with peers was represented by the mean of three indicators: “How likely is it that you would discuss environmental issues with your peers?,” “How likely is it that you would talk to your peers about the things that you do to help the environment?,” and “How likely is it that you would tell your peers about ways that people can help the environment?” The internal reliability of the construct was good with alphas above 0.82 at each survey point (see Table 1 for M and SD at each survey point). Two further indicators measured less passive group environmental behavior: “How likely is it that you would correct one of your peers who is harming the environment (e.g., dropping litter)?” and “How likely is it at that you would do things to help the environment (e.g., picking up litter) when you are together with your peers?” (see Table 1 for M and SD at each survey point).

###### Parents’ pro-environmental behavior

The behavior measure was repeated at the end of each survey, but with “parents/caregivers” as the subject. The participants’ environmental dialog with their parents should not change as a result of the intervention and was thus used as a control to examine the influence of the intervention on the participants environmental dialog with their peers. The internal reliability (alpha) of the construct was above 0.92 at each survey point (see Table 1 for M and SD at each survey point).

Analyses of the significance of the differences between surveys, and between participants’ behavior with peers and with parents/caregivers, was determined using paired *t*-tests. Bivariate (Pearson’s) correlations between the measured variables were determined. The significance of differences between correlations was determined using a Fisher *r*-to-*z* transformation. The significance of the hypotheses was confirmed using a Bonferroni-Holm correction. Other statistical analyses were performed using SPSSv27.

#### Study 1 results

##### Personal biospheric values and behaviors

In the New Zealand study (Table 1), biospheric values were strongly endorsed by the participants prior to the intervention, and this strong endorsement of biospheric values did not change significantly up to 3 months post-intervention [*t*(62) = 0.145, *p* = 0.885]. The self-reported frequency of personal pro-environmental behavior was also consistent during this time [*t*(62) = 0.957, *p* > 0.342].

The participants’ biospheric values were moderately positively correlated with their personal pro-environmental behaviors, both pre-intervention (Table 2) and post-intervention (Table 3), thus supporting Hypothesis 1.

**TABLE 2:** Pre-intervention correlations (NZ).

	Personal values	Peers’ values	Personal PEB	Enviro dialog peers	Enviro dialog parents	Challenge peers
Personal biospheric values	1					
Perceived peers’ biospheric values	0.44***	1				
Personal PEB frequency	0.23*	−0.09	1			
Environmental dialog with peers	0.38**	0.16	0.42***	1		
Environmental dialog with parents	0.39**	0.10	0.41***	0.71***	1	
Challenge peers harming environment	0.48***	0.19	0.37**	0.49**	0.51***	1
Help environment when with peers	0.36**	0.08	0.37**	0.52***	0.54***	0.64***

PEB, pro-environmental behavior. *p < 0.05, **p < 0.01, ***p < 0.001.

**TABLE 3:** Post-intervention correlations (NZ).

	Personal values	Peers’ values	Personal PEB	Enviro dialog peers	Enviro dialog parents	Challenge peers	Help enviro w/peers
Personal biospheric values	–	0.37**	0.48***	0.49***	0.52***	0.52***	0.54***
Perceived peers’ biospheric values	0.57***	–	0.17	0.50***	0.40***	0.33**	0.37**
Personal PEB frequency	0.32*	0.26*	–	0.32**	0.37**	0.43***	0.48***
Environmental dialog with peers	0.57***	0.47***	0.31*	–	0.79***	0.61***	0.60***
Environmental dialog with parents	0.49***	0.29*	0.42**	0.81***	–	0.45***	0.49***
Challenge peers harming environment	0.38**	0.41**	0.48***	0.63***	0.56***	–	0.72***
Help environment when with peers	0.57***	0.48***	0.37**	0.67***	0.56***	0.73***	–

Post-intervention above the diagonal, and 3 months post-intervention below the diagonal. PEB, pro-environmental behavior. *p < 0.05, **p < 0.01, ***p < 0.001.

##### Peers’ biospheric values and behaviors

The participants’ personal biospheric values were moderately positively correlated with their perception of their peers’ biospheric values (Tables 2, 3), supporting Hypothesis 2. In addition, as found in previous research with adults (Bouman et al., 2020), the participants believed that their peers’ biospheric values were significantly lower than their own [*t*(76) = 5.10, *p* > 0.001], and this finding remained stable post-intervention [*t*(74) = 4.28, *p* > 0.001] and 3 months post-intervention [*t*(62) = 4.86, *p* > 0.001] (Table 1). Thus, Hypothesis 3 was also supported. Hypothesis 4 was not supported, as the participants’ perception of their peers’ values did not change significantly over the course of the study [*t*(62) = 5.98, *p* = 0.552] (Table 1).

Pre-intervention, the participants’ perception of their peers’ biospheric values was not correlated with either their personal pro-environmental behavior nor their group pro-environmental behaviors (Table 2). However, by 3 months post-intervention, a significant correlation had developed between their perception of their peers’ biospheric values and both their personal and group pro-environmental behaviors (Table 3). These changes in correlation were significantly different in the case of “personal pro-environmental behavior” (*z* = 2.05, *p* = 0.038), “environmental dialog with peers” (*z* = 2.01, *p* = 0.042), and “helping the environment when with peers” (*z* = 2.55, *p* = 0.010), but not significantly different in the case of “challenging peers who are harming the environment” (*z* = 0.71, *p* = 0.478).

We observed a significant increase in the likelihood that participants would engage in environmental dialog with their peers between pre-intervention and 3 months post-intervention [*t*(61) = 2.91, *p* = 0.005]. In tandem, we observed no change over time for the control variable “environmental dialog with parents” [*t*(60) = 0.446, *p* = 0.657], a finding which supports the likelihood that the observed increase in environmental dialog with peers was a result of the intervention rather than chance. We observed no significant change over the course of the study in the other measured group behaviors, those being challenging peers who are harming the environment [*t*(61) = 0.773, *p* = 0.443] and helping the environment when with peers [*t*(61) = 1.81, *p* = 0.076].

### Study 2 (the Netherlands)

#### Study 2 method

##### Participants and design

In the Netherlands an environmental education program was delivered by Groninger Landschap, a nature organization that focuses on the preservation and conservation of the regional natural and cultural environment. The half day education program was primarily aimed at making school children aware of the animals (birds and/or mammals) living in the region, which it aimed to achieve through group-based work and activities. Although the intervention did not explicitly discuss values, we hypothesized that through actively engaging in nature activities together, children would learn about classmates’ endorsement of biospheric values. Children from seven schools in the Groningen district participated in the education programme. Participation in the data collection was open to all children in the programme.

After parents (who were informed at least a week before the intervention and study) and children agreed to the informed consent, the children were presented with the survey (in Dutch). The survey first asked the children to generate a personal code so that the researchers could link the answers provided at the first and second survey points. We attempted to collect two waves of data pre-intervention, to act as a control, but only a few of the participating schools were open to this. The surveys were administered on pen and paper about a week prior to beginning the intervention and immediately afterward. The study was approved by (the Ethical Committee Psychology of the University of Groningen).

By analyzing standard deviations across individual constructs, and by examining responses to reverse coded variables, disengaged cases were identified and eliminated from the study. A criterium for inclusion in the investigation was that both surveys were fully completed. The final number of cases analyzed was 198, and 49 cases were omitted due to lack of completion or disengagement. Ninety-four of the participants were female, ninety-eight were male, and six did not identify their gender. The average age of participants was 10.6 years old, with an age range of 9 to 12 years.

##### Measures

The measures were presented in the survey in the following order:

###### Personal biospheric values

We adapted Bouman et al. (2018) Environmental Portrait Values Questionnaire (E-PVQ) to measure children’s values. In this version, we rephrased items to make them more comprehensible for children and shortened the scale to 10 items to not overburden participants with long questionnaires. Each item reflected a statement, for which children had to indicate how important that statement was to them personally on a 10-point scale (1 “*not at all*” to 10 “*of utmost importance*”). Biospheric values were measured with four items, namely: “How important is it to you… that we are able to live together with animals and plants without disturbing their lives,” “… to feel connected to nature, that you belong to nature,” “… that people take care of nature and the environment,” and “… that we ensure that nature will not be polluted.” For the four biospheric values indicators the internal reliability (alpha) of the construct was greater than 0.81 at each survey point.

As with the New Zealand investigation, we also asked participants to complete a short basic human values scale to indicate their own values. The indicators used were “I find it very important to respect and protect nature” (biospheric values), “…that everybody has the same opportunities and is treated equally” (altruistic values), “… to have fun and enjoy life” (hedonic values), and “… to be popular, have a lot of possessions, and to determine what others should do” (egoistic values). The short questionnaire also used a ten-point scale and, as for the full scale, the participants were instructed to vary their scoring for each indicator as much as possible. At each survey point we found that the short scales measuring the participants’ own values were highly correlated with the corresponding long scales (*r*s > 0.79) and can thus be considered valid indicators of basic human values (see Table 4 for M and SD at each survey point). For the sake of consistency, and to be able to compare personal values with perceived values of peers, only the findings from the short value scales are presented in this article.

**TABLE 4:** Values and behavior at each survey point (NL).

	Pre-intervention		Post-intervention	
	**Mean**	**SD**	**Mean**	**SD**
Personal biospheric values	8.53	1.58	8.61	1.74
Perceived friends’ biospheric values	8.14	1.60	8.39	1.70
Would turn off lights when leaving room	8.24	2.14	8.37	1.98
Likelihood of picking up others’ litter	5.08	2.70	5.08	2.86
Likelihood of challenging classmate littering	6.01	2.86	6.01	2.82
Would attend an environmental event	6.58	2.77	7.08	2.73
Would invite peers to an environmental event	4.12	3.09	4.88	3.32
Nature protection concern	8.39	1.47	8.75	1.44

All scores out of ten.

###### Personal pro-environmental behavior

To measure a potential behavior spillover effect, we also asked participants to indicate how often they engaged in the following actions: “If I leave a room, I turn off the lights” and “Picking up litter from the street?”. Responses were measured on a ten-point scale, with 1 being “never” and 10 “always.”

###### Friends’ biospheric values

The short personal values scale was adapted to the group level, asking about “my friends at school” instead of “I” [e.g., “My friends at school find it very important to respect and protect nature” (biospheric values)]. Significantly, the indicators in the Netherlands study pertained to “friends,” whereas the indicators in the New Zealand study pertained to “peers.” Friends are likely to be more influential than peers (Long et al., 2014), and thus may be more likely to cause changes to participants’ values and behaviors. Differences between the participants’ personal biospheric values and their perception of their friends’ biospheric values, and the difference between these values at the different survey points, were determined.

###### Friends’ pro-environmental behavior

Whereas in the New Zealand study environmental dialog with peers was measured by asking the participants about the likelihood that they would engage in discussion about environmental issues and environmental behaviors with their peers, in the Netherlands study this was measured by asking participants “How likely is it that you would invite a friend to an environmental event?” As in the New Zealand study, less passive group environmental behavior was measured, in this case by asking “How likely is it that you would correct one of your peers who is dropping litter?” Participants were also asked “Would you attend an environmental event?,” which may be considered a group behavior. Responses to these behavior indicators were measured on a ten-point Likert scale, with 1 being “never” and 10 “always.”

###### Concern for nature

The participants’ concern for nature protection was determined by three indicators on a scale of one to ten (1 “*not at all*” to 10 “*of utmost importance*”): “How important is it to protect the (birds/mammals/nature) in our region?” (alpha > 0.81).

##### Analyses

Differences at each survey point were determined using paired *t*-tests. Bivariate (Pearson’s) correlations between the measured variables were determined. The significance of differences between correlations was determined using a Fisher *r*-to-*z* transformation. The significance of the hypotheses was confirmed using a Bonferroni-Holm correction. Other statistical analyses were performed using SPSSv27.

#### Study 2 results

##### Personal biospheric values and behaviors

As in the New Zealand study, biospheric values were strongly endorsed by the children in the Netherlands study prior to the intervention (Table 4), and the strength of this endorsement did not change significantly post-intervention [*t*(196) = 0.836, *p* = 0.404]. Personal pro-environmental behavior, as indicated by the likelihood that participants would turn out the light when leaving a room, was high pre-intervention (Table 4) and did not change significantly over the course of the study [*t*(192) = 1.07, *p* = 0.287]. The likelihood of picking up other people’s litter was less common (Table 4), and likewise did not change post-intervention [*t*(190) = 0.055, *p* = 0.957]. Although the participants’ concern for nature protection was high pre-intervention (Table 4), it nonetheless increased significantly after the intervention [*t*(195) = 4.55, *p* < 0.001]. The participants’ personal biospheric values were positively correlated with their pro-environmental behaviors (Table 5), supporting Hypothesis 1.

**TABLE 5:** Correlations before and after the intervention (NL).

	Own values	Friends’ values	Invite peers	Challenge peers	Pick up litter	Attend event	Lights off	Nature concern
Own biospheric values	–	0.68***	0.15**	0.31***	0.30***	0.13*	0.37***	0.66***
Perceived friends’ biospheric values	0.61***	–	0.13*	0.22***	0.16**	0.10	0.29***	0.49***
Invite friends to an enviro event	0.11	0.12	–	0.36***	0.38***	0.39***	0.13*	0.27***
Challenge classmate littering	0.34***	0.22**	0.33***	–	0.55***	0.24***	0.25***	0.30***
Pick up other’s litter	0.27***	0.24***	0.30***	0.51***	–	0.27***	0.17**	0.34***
Attend an enviro event	0.25***	0.16*	0.38***	0.18**	0.28***	–	0.03	0.28***
Turn off lights when leaving room	0.31***	0.24***	0.10	0.34***	0.29***	0.19***	–	0.25***
Nature protection concern	0.69***	0.45***	0.19**	0.31***	0.28***	0.27***	0.31***	–

Pre-intervention correlations below the diagonal, and post-intervention correlations above. *p < 0.05, **p < 0.01, ***p < 0.001.

##### Friends’ biospheric values and group behaviors

Supporting Hypothesis 2, the participants’ personal biospheric values were positively correlated with their perception of their friends’ biospheric values (Table 5). Supporting Hypothesis 3, and mirroring our finding in the New Zealand study, we found that the participants believed their friends’ biospheric values to be significantly weaker than their own biospheric values, both before [*t*(197) = 4.00, *p* < 0.001] and after [*t*(196) = 2.43, *p* = 0.016] the intervention (Table 4). However, unlike in the New Zealand study, we found that there was a significant increase in the participants’ perception of their friends’ biospheric values after the intervention [*t*(76) = 5.10, *p* > 0.001], thus supporting Hypothesis 4.

As regards group behavior, the participants indicated post-intervention that they would be more likely to want to attend a similar group environmental education event [*t*(190) = 2.67, *p* = 0.008], and more likely to invite a friend to such an event [*t*(190) = 4.11, *p* < 0.001]. The intervention had no significant impact on whether the participants would be likely to challenge friends who were littering [*t*(189) = 0.034, *p* = 0.973].

## General discussion

Our research found that children generally care about protecting the environment and value the natural world, as demonstrated by their strong endorsement of biospheric values. These values were positively related to pro-environmental behaviors that are particularly relevant for children, such as turning off lights and not littering. Children often undertake behaviors not of their own volition, so it is useful to confirm that their personal pro-environmental behaviors are related to valuing the natural environment, and not just governed by external motives such as school rules and parental expectations.

Group pro-environmental behaviors, such as discussing environmentalism with peers or challenging those who harm the environment, were also positively related to children’s personal biospheric values. Since children’s personal values are in part shaped by the behaviors and values of their friends and peers (Benish-Weisman et al., 2022), these group behaviors may be particularly important during this critical age when personal values are becoming more fixed. Environmental interactions with friends and peers may normalize valuing the environment and lead to further endorsement of biospheric values through socialization and education processes. Increased endorsement of biospheric values may in turn result in increased group pro-environmental behaviors as a way of expressing one’s values.

The perception of what friends and peers value may be a significant factor in strengthening or weakening children’s biospheric values. As found previously with adults (Bouman et al., 2020; Wang et al., 2021), there was a positive correlation between personal biospheric values and the perception of friends’/peers’ biospheric values in both samples of children. This correlation suggests that increasing children’s perception of their peers’ biospheric values may be important in developing these values in individuals, that is, believing that friends and peers value the natural environment may lead to an individual’s further endorsement of biospheric values. As the correlation was present in our study for both “peers” (New Zealand) and “friends” (the Netherlands), this may indicate that the effect is due to socialization rather than selection, as “friends” are selected, but “peers” not necessarily so (Brechwald and Prinstein, 2011).

The perception of friends’/peers’ biospheric values was in addition positively related to the participants’ pro-environmental behaviors, although in the New Zealand study this correlation only developed after the intervention. This correlation suggests that exposure to peers’ values in an environmental education setting can help develop connections that may influence both personal and group environmental behavior.

We found that, like adults (Bouman et al., 2020), children underestimate the biospheric values of their friends and peers. This may reduce their motivation to endorse these values and engage in pro-environmental behaviors. We propose that interventions that increase children’s perception of their peers’ biospheric values may strengthen their own values through socialization pathways. Children who want to fit in with their social group may change their values and behaviors if it helps them feel more congruent within the group. However, if educators do not address children’s underestimation of their peers’ biospheric values, there is a risk of creating a negative feedback loop that diminishes the child’s biospheric values over time.

Our study of Dutch children found that group environmental education programmes may be effective in partially correcting the underestimation of the group’s biospheric values. Although the study with New Zealand children did not demonstrate a significant change in the perception of their peers’ biospheric values after the education programme, there was a significant increase in environmental interactions between peers after the intervention, which may help children better understand their friends’ and peers’ biospheric values over time.

### Limitations

It is important to note that the interventions we investigated were not designed to influence the participants’ perception of their peers’ biospheric values. Rather, the interventions were designed to influence the participants’ own biospheric values directly, but in situations wherein the presence of peers could have an influence on these values. Accordingly, our studies and analyses could be regarded conservative tests of our Hypothesis 4 and potentially larger effects could have been obtained with educational programs that more directly address the observed value underestimations.

The significance of our findings is limited by the relatively small sample sizes employed in these studies. The small sample size in the New Zealand study was somewhat alleviated by comparing the participants’ perceptions of their parents to their peers, as their perception of their parents should not change significantly as a result of the intervention, as found.

The self-reported nature of the data is a limitation, insomuch as self-reported behavior is not considered to be a good indicator of actual behavior (Kormos and Gifford, 2014), although we are unsure whether this would hold true for children. The pro-environmental behavior measures we employed should be prone to ceiling and floor effects, but this was not evident in the data, which raises some questions about the accuracy of the self-reported behavior data. However, it may be expected that children’s behavior is less prone than adult’s behavior to ceiling/floor effects, as children are likely to have more variable behavior, due to their behavior being more subject to external factors, such as rules or parental expectations.

With datasets from two different countries it is tempting to investigate cultural differences. However, since our samples were not representative, and different interventions were investigated in each country, we do not feel it is appropriate to make cultural comparisons. That said, it is important to note that we did find similar results across countries and educational programs, which suggest the universality and generalisability of the studied relationships.

### Future research

There is a lack of research in general that has addressed the development of biospheric values in children, and future research is needed to determine the influence of different factors from childhood through to early adulthood. Our research has found that children underestimate the extent to which their peers endorse biospheric values, and therefore it would be useful to further investigate why this occurs, and the long-term impacts of rectifying this underestimation. This is particularly salient given that exposure to the values of those people who are closest to us has been found to have a significant effect on our values and pro-environmental behaviors later in life (Molinario et al., 2020). In addition, Molinario et al. (2020) found that extraordinary childhood experiences of nature are important for the development of biospheric values, and pro-environmental behaviors especially, in adulthood. This raises the question of whether ordinary environmental education programmes are effective in their goals. Longitudinal studies would help determine whether the effects of interventions that change the perception of the group values are long lasting and eventually strengthen the participants’ own biospheric values. In addition, the efficacy of shorter-term educational programmes and events compared to sustained, long-term educational programmes merits investigation.

It would be useful for future research to examine the cause-and-effect relationship between group biospheric values and group environmental dialog, and to determine the effect of each on children’s own biospheric values, and their perception of the group biospheric values, in the longer term. There are many different types of intervention that are employed to encourage environmentalism in children, and it would be beneficial to determine if those interventions that promote group dialog are more effective than those that do not.

## Conclusion

In conclusion, this study provides a deeper understanding of the relationship between children’s biospheric values and pro-environmental behaviors and how group environmental education interventions may affect these relationships. Our findings suggest that children’s biospheric values are positively related to their pro-environmental behaviors, and that their perception of their peers’ biospheric values may play an important role in the development and strength of their own values. The study also highlights the importance of addressing the underestimation of peers’ values, as this may reduce motivation to engage in pro-environmental behaviors. The results provide support for the idea that group environmental education interventions can be effective in increasing some group pro-environmental behaviors and in helping children better understand their peers’ values. Future research should continue to explore different types of interventions and study designs to further our understanding of how to effectively influence the development of children’s biospheric values and pro-environmental behaviors.

## Data availability statement

The datasets presented in this article are not readily available because due to the age of the participants, the ethics approval for this study has restricted the sharing of data outside of the immediate research group. Requests to access the datasets should be directed to TK, tim.kelly@canterbury.ac.nz.

## Ethics statement

The studies involving humans were approved by the University of Canterbury’s Educational Research Human Ethics Committee and Ethical Committee Psychology, University of Groningen. The studies were conducted in accordance with the local legislation and institutional requirements. Written informed consent for participation in this study was provided by the participants’ legal guardians/next of kin.

## Author contributions

TK: Conceptualization, Data curation, Formal analysis, Investigation, Methodology, Project administration, Writing – original draft. TB: Conceptualization, Data curation, Investigation, Methodology, Writing – original draft. SK: Writing – review and editing. FW: Writing – review and editing, Data curation, Investigation. RG: Writing – review and editing.
